# Point of care testing for urinary tract infection in primary care (POETIC): protocol for a randomised controlled trial of the clinical and cost effectiveness of FLEXICULT™ informed management of uncomplicated UTI in primary care

**DOI:** 10.1186/s12875-014-0187-4

**Published:** 2014-11-25

**Authors:** Janine Bates, Emma Thomas-Jones, Timothy Pickles, Nigel Kirby, Micaela Gal, Emily Bongard, Kerenza Hood, Nicolas Francis, Paul Little, Michael Moore, Kate Rumsby, Carlos Llor, Curt Burgman, Theo Verheij, David Cohen, Mandy Wootton, Robin Howe, Christopher C Butler

**Affiliations:** South East Wales Trials Unit (SEWTU), School of Medicine, Cardiff University, 7th Floor Neuadd Meirionnydd, Heath Park, Cardiff, CF14 4XN UK; Institute of Primary Care & Public Health, School of Medicine, Cardiff University, 5th Floor Neuadd Meirionnydd, Heath Park, Cardiff, CF14 4XN UK; Department of Primary Medical Care, University of Southampton, Aldermoor Close, Southampton, SO16 5ST UK; Primary Care Centre Jaume I, University Rovira i Virgili, Felip Pedrell, 45-47, 43005 Tarragona, Spain; Julius Center for Health Sciences and Primary Care, UMC Utrecht, PO Box 85500, Utrecht, GA 3508 Netherlands; Health Economics and Policy Research Unit, University of Glamorgan, Faculty of Health, Sports & Science, Pontypridd, CF37 1DL UK; Specialist Antimicrobial Chemotherapy Unit, Public Health Wales Microbiology Cardiff, University Hospital Wales, Heath Park, Cardiff, CF14 4XW UK

**Keywords:** Urinary Tract Infection, Primary care, Adult women, Point-of-care-test, Near-patient testing, Antibiotic resistance, Cost effectiveness

## Abstract

**Background:**

Urinary tract infections (UTI) are the most frequent bacterial infection affecting women and account for about 15% of antibiotics prescribed in primary care. However, some women with a UTI are not prescribed antibiotics or are prescribed the wrong antibiotics, while many women who do not have a microbiologically confirmed UTI are prescribed antibiotics. Inappropriate antibiotic prescribing unnecessarily increases the risk of side effects and the development of antibiotic resistance, and wastes resources.

POETIC is a randomised controlled trial of a Point Of Care Test (POCT) (Flexicult™) guided UTI management strategy for use in primary care, which may help General Practitioners more effectively decide both *whether* or not to prescribe antibiotics, and if so, to select the *most appropriate* antibiotic.

**Methods/design:**

614 adult female patients will be recruited from four primary care research networks (Wales, England, Spain, the Netherlands) and individually randomised to either POCT guided care or the guideline-informed ‘standard care’ arm. Urine and stool samples (where possible) will be obtained at presentation (day 1) and two weeks later for microbiological analysis. All participants will be followed up on the course of their illness and their quality of life, using a 2 week self-completed symptom diary. At 3 months, a primary care notes review will be conducted for evidence of further evidence of treatment failures, recurrence, complications, hospitalisations and health service costs.

The primary objective is to compare appropriate antibiotic use on day 3 between the POCT and standard care arms using multi-level logistic regression to produce an odds ratio and associated 95% confidence interval. Costs of the two management approaches will be assessed in terms of the primary outcome.

**Discussion:**

Although the Flexicult™ POCT is used in some countries in routine primary care, it’s clinical and cost effectiveness has never been evaluated in a randomised clinical trial. If shown to be effective, the use of this POCT could benefit individual sufferers and provide evidence for health care authorities to develop evidence based policies to combat the spread and impact of the unprecedented rise of infections caused by antibiotic resistant bacteria in Europe.

**Trial registration number:**

ISRCTN65200697 (Registered 10 September 2013).

## Background

Each year, about 10% of adult women experience a urinary tract infection (UTI) and about 60% experience a UTI at some point in their lives [1,2]. Recurrences are common, with nearly half going on to experience a subsequent UTI within a year. UTI accounts for about 15% of antibiotics prescribed in primary care, and between 34% and 60% of patients treated with an antibiotic do not have a microbiologically proven UTI and 25% of those with a positive urine culture are not prescribed antibiotics.

For those who are likely to benefit, antibiotics have been shown to reduce symptom duration [3]. However, some do not benefit from antibiotics and as these carry risks of side effects, increased antibiotic resistance and costs, unnecessary antibiotic use should be minimised. In a systematic review and meta-analysis of five studies of urinary tract bacteria that included 14 348 participants, the pooled odds ratio (OR) for resistance was 2.5 (95% CI 2.1 to 2.9) within two months of antibiotic treatment and 1.33 (95 % CI 1.2 to 1.5) within 12 months [4].

Antibiotic resistant UTIs are common in primary care, with recent antibiotics the greatest single risk factor for a resistant compared to a sensitive infection [5]. Resistant infections are associated with longer illness duration, increased re-consultations, and increased health care costs [6,7].

Current strategies to predict bacteriological UTI in adult women need refining so that more women who will benefit from antibiotic treatment are prescribed them, antibiotic treatments are better targeted to the sensitivity of the infecting organisms, and antibiotics are prescribed less often for women who will not benefit. There is thus an urgent need to support General Practitioners in deciding both *whether* or not to prescribe antibiotics and, if so in selecting the *most appropriate* antibiotic. Accurate diagnosis and rational treatment is required to minimise acute suffering and help prevent recurrences.

The POETIC trial is an evaluation of a Point of Care Test (POCT) guided UTI management strategy for use in adult women presenting in primary care with suspected uncomplicated UTI. The POCT that will be evaluated is called Flexicult™ and is a simple point of care culture procedure, and provides clinicians, at the point of care, within 24 hours, with a microbiological diagnosis of bacterial UTI by quantifying bacteria in the urine sample (or not) and determining sensitivities of any identified pathogen to the antibiotics most commonly used for UTI in primary care [8].

POETIC aims to determine the clinical effects and costs of this optimised POCT guided diagnostic and treatment strategy for symptoms of uncomplicated UTI on the overall appropriateness of antibiotic prescribing when compared to a “practice based on best available local guidelines” strategy. The POETIC trial will determine whether or not the POCT aids clinicians to more appropriately prescribe antibiotics for uncomplicated UTI’s i.e. minimise the use of antibiotics for women where no bacterial infection is identified, and ensure the narrowest spectrum antibiotic appropriate to the sensitivity of the infecting organisms is prescribed when a bacterial infection is identified, and whether or not the strategy is cost effective.

## Methods/design

### Trial design

POETIC is a two-arm, individual patient randomised controlled trial aiming to quantify the effects and costs of an optimised POCT guided diagnostic and treatment strategy for symptoms of uncomplicated UTI on the overall appropriateness of antibiotic prescribing when compared to a “practice based on best available local guidelines” strategy. The research will be implemented in primary care research networks in the four participating European countries (Wales, England, the Netherlands and Spain).

The trial will randomise 614 eligible women to either the POCT guided management strategy, or to a standard UTI management strategy based on best local guidelines.

### Setting

This multinational trial will be implemented in primary care research networks based in Wales, England, Spain and the Netherlands. Each centre will recruit adult women with symptoms attributable to UTI, over the age of 18 years, from their affiliated primary care sites.

### Trial intervention

Participants randomised to the experimental intervention arm will have their treatment guided by the Flexicult™ POCT. This POCT is a point of care culture-based approach and involves fresh urine being placed on a special agar plate and the excess urine poured off. The plate is then placed in a simple desktop incubator within the practice, and read approximately 24 hours later.

Patients can only be included in the POCT arm if the clinician/nurse is able to read the plate 24 hours later. The plate is divided into six segments. The largest compartment contains culture medium alone, and the other five segments contain culture medium with impregnated with different antibiotics with specific concentrations commonly used for treating UTI in primary care. This allows for assessment of bacterial growth (quantification), evaluation of the species present, and assessment of resistance to the antibiotics in each of the plate segments.

The Flexicult™ system has been used in primary care settings in Denmark for approximately 10 years. POETIC will use new Flexicult™ plates that have been developed by the manufacturer and CE marked (Statens Serum Institut, SSI) to include the antibiotics that are most commonly used in the three participating regions (UK -England and Wales, Spain and the Netherlands) (see Table 1 below):

**Table 1:** **Antibiotics included in Flexicult™ plate for each region**

**UK**	**Netherlands**	**Spain**
Trimethoprim	Trimethoprim	Fosfomycin
Nitrofurantoin	Nitrofurantoin	Nitrofurantoin
Amoxicillin/clavulanate	Amoxicillin/clavulanate	Amoxicillin/clavulanate
Ciprofloxacin	Ciprofloxacin	Ciprofloxacin
Cephalexin	Amoxicillin	Cefuroxime

Clinicians will be provided with training in the management of UTI using the Flexicult™ approach. Two treatment approaches will be available to participating clinicians for patients randomised to this arm:

1. Provide patients with advice about symptomatic management of their UTI for approximately 24 hours and arrange a mechanism for contacting the patient in approximately 24 hours in order to arrange provision of a prescription for antibiotics, or not, as appropriate, based on the results of the Flexicult™ POCT test.
2. Provide patients with a prescription for antibiotics to start immediately and arrange a mechanism for contacting the patient approximately 24 hours later in order to advise the patient to continue taking the antibiotics, stop taking the antibiotics, or switch to an alternative antibiotic, as appropriate, based on the results of the Flexicult test.

Clinicians will be encouraged to adopt the first approach for patients with milder symptoms and/or for those that would be happy/would prefer to wait 24 hours for a more definitive diagnosis, and the latter approach for those with more severe symptoms or who are not happy to delay initiation of antibiotics.

### Control arm –standard care

Patients randomised to the control arm will receive standard care informed by national guidelines. Clinicians will receive a summary of national guidelines on the management of uncomplicated UTI in primary care (UK, Spain and the Netherlands). Participating clinicians will be provided with the relevant summary for their country, and training in best practice based on their national guidelines. The management of patients who are randomised to the standard care arm will therefore be based upon the management decisions of clinicians who have received training in best practice and a summary of their national guideline(s). Management decisions in these patients randomised to guideline informed usual care will *not* be guided by the POCT.

### Primary objective

The primary outcome will be appropriate antibiotic use on day 3 (with day 1 being the day that the patient consulted with their primary care clinician). For women who are shown to have a UTI. Appropriate antibiotic use on day 3 will be defined as use of an antibiotic for which the isolated pathogen has been shown to have *in vitro* sensitivity (based on laboratory analysis). For women who do not have a UTI, appropriate antibiotic use on day 3 will be defined as no antibiotic use on this day. In the UK the definition of a UTI is defined for the POETIC trial as ≥10^5^ colony forming units per millilitre (cfu/ml) of a pure/predominant recognised uropathogen, however the varying definitions across the countries will be considered.

### Secondary objectives

The secondary objectives are to compare intervention arms with regard to:

- Antibiotic choice in relation to presence of infection and organism susceptibility and antibiotic spectrum
- Dose and duration of antibiotic prescribed
- Proportion of patients receiving antibiotic prescription
- Adherence to national prescribing guidelines
- Symptoms/recovery
- Recurrence of UTI (within a three month period)
- Patient satisfaction with management
- Antibiotic resistance in urine and stool samples at two week follow up
- Direct/indirect costs
- Cost effectiveness

### Ethical and governance approval

This trial protocol was approved by the Research Ethics Committee (REC) For Wales recognised by the United Kingdom Ethics Committee Authority (UKECA) and also approved by the relevant local Governance Committees in the Netherlands and Spain.

All sites within the UK received Research & Development approval from the appropriate Health Boards and Trusts before commencing trial. For Spain, approval was obtained by the Institut d‘Investigació d’Atenció Primària Jordi Gol i Gurina, Barcelona. For the Netherlands, approval was obtained from Medisch Ethische Toetsingscommissie of the Universitair Medisch Centrum Utrecht (University Medical Center Utrecht).

### Trial procedures

#### Primary care sites/GP practice recruitment

The trial centres have been selected on the basis of having well-established primary care research networks and representing contrasting European cultures and healthcare systems. Each centre will recruit around 10 general practices to participate in the trial. Practices will be selected on the basis of recruitment potential, with larger practices and practices which have previous experience in recruiting participants during routine consultations.

All participating practices will receive face-to-face training with additional online resources, via a bespoke POETIC website (www.POETIC-study.co.uk), in use of the POCT strategy. This will include sample collection and preparation, use of the POCT, interpretation of the POCT results, data collection and storage, health and safety issues and management strategies (how to use the results of the POCT in line with best evidence and local guidelines).

#### Microbiology laboratory recruitment

The participation of any primary care site in recruitment to the trial requires the support and participation of a research microbiology laboratory in each of the participating countries, to which the site sends urine and stool samples for the purposes of the POETIC trial.

For sites in Wales and England, samples will be sent to the Specialist Antimicrobial Chemotherapy Unit (SACU), Public Health Wales Microbiology Cardiff, at University Hospital of Wales. In The Netherlands, samples will be sent to the Department of Medical Microbiology of the Universitair Medisch Centrum Utrecht. In Spain, the samples will be sent to the Microbiological Departments of the Hospitals Ramón y Cajal (Madrid), Joan XXIII (Tarragona), and Bon Pastor (Barcelona). All laboratories will be provided with a POETIC microbiology manual and standard operating procedures.

### Participant recruitment

**Figure 1:**
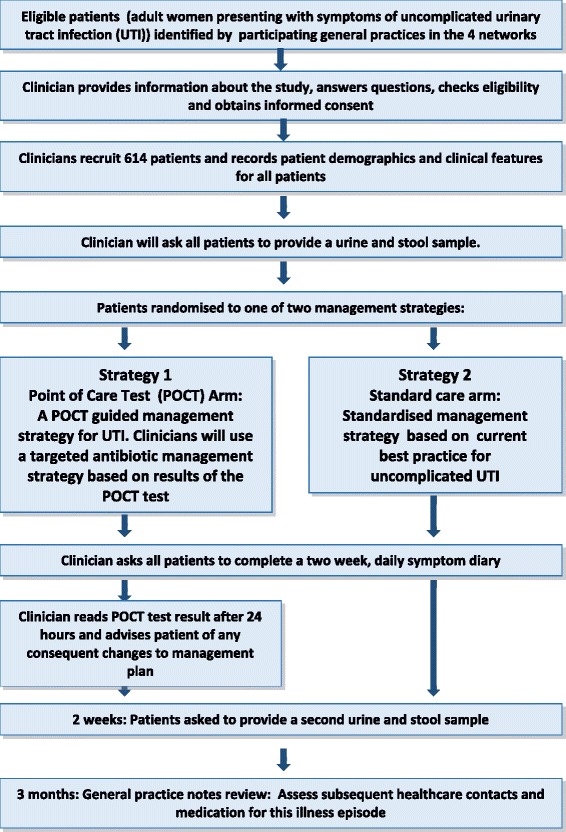
**Trial schema and participant flow diagram.** Figure 1 provides an overview of the POETIC trial and participant flow process.

The recruitment process is summarised in Figure 1.

#### Registration and consent

Participating clinicians (GPs or nurse prescriber) will identify eligible patients during routine general practice consultations. The participating clinician will assess eligibility, provide potential participants with a verbal description of the trial, and if the patient is interested, will provide them with a comprehensive participant information leaflet (PIL). All potential participants will be given sufficient time to read the PIL, ask questions, and consider participation before being asked to provide informed consent. Women who consent to take part will be asked to sign a consent form, which will also be signed by the clinician who is taking consent. Copies of the consent form will be kept in the site file, and sent securely to the trial team in each centre.

All participating practices will be asked to keep an anonymous screening log of all ineligible and eligible but not consented/not approached patients. These will be used to detect any selection bias.

Participants will be informed that they have the right to withdraw consent for participation in the POETIC trial at any time and that their clinical care will not be affected at any time by declining to participate or withdrawing from the trial.

#### Inclusion criteria

Women aged 18 years and older presenting to primary care with at least one of three key urinary tract symptoms (dysuria, urgency including nocturnal and frequency that have been present for up to 14 days) and where the clinician suspects uncomplicated UTI will be eligible to take part. Patients should be able to provide written informed consent and be willing to complete the patient diary.

#### Exclusion criteria

Women with one or more of the following will not eligible for inclusion:

- Terminally ill
- Currently receiving treatment for life-threatening cancer (basal cell carcinoma, for example, excluded)
- Other severe systemic symptoms, such as high fever, renal angle pain, rigors.
- On long-term antibiotic treatment or have received antibiotics for urinary tract infection within the past four weeks
- Has had bladder surgery (including cystoscopy) within the past four weeks
- Known or likely to have significant immune compromise (i.e. known immunodeficiency state, on long-term corticosteroid or chemotherapy treatment, insulin dependent diabetes)
- Known functional or anatomical abnormalities of the genitourinary tract
- History of pyelonephritis
- Known pregnancy
- Unable to provide a urine sample on the day of first presentation

The clinician will need to ensure the participant provides a mid-stream urine (MSU) sample before leaving the practice. Patients can only be included in the POCT arm if the clinician is present to read the plate 24 hours later.

### Urine and stool sample collection

A urine (required for participation) and stool (if possible) sample will be collected from the participant on day 1 (day of recruitment) and at day 14 and analysed in the research laboratories for presence of bacteria and any resistance profiles. Patients who do not wish to provide stool samples but are happy to participate in all other aspects of the trial will be eligible for recruitment, as the stool samples are not necessary for answering the primary aim of the trial.

Participants will be provided with written instructions on how to collect the urine and stool samples. Urine samples will be collected using a mid-stream urine collection kit (Peezy MSU collection kit) to ensure a clean catch. Stool samples will be collected using an ‘Easy sampler’ kit. Participants will be asked to return samples to the laboratory within 24 hours of collection.

If the clinician requires a urine sample to be sent as part of their clinical management, and there is insufficient urine for both clinical and research samples, the clinical sample will take priority.

Urine samples, from both trial arms, will be transferred to a container containing boric acid for transport to the research laboratory, to arrive at the laboratory within 24 hours for identification and testing of antimicrobial resistance. Results from the research microbiology laboratories will not routinely be disclosed to participating clinicians or patients in either trial arm, but will be available upon request in case of clinical deterioration.

### Data collection

#### Baseline case report form (CRF) – all participants

##### Eligibility

An eligibility CRF will be completed for all consented participants to ensure that they meet the eligibility criteria set out in the protocol. The clinician will then sign the form to confirm eligibility.

##### Randomisation

The randomisation and allocation of eligible participants will be undertaken as part of the data collection process. Whilst completing the baseline CRF, clinicians will be asked to randomise the patient using a password protected POETIC randomisation website. Each practice will be allocated unique login details to the website. The clinician will enter the relevant data into the website, including the required stratifying and balancing variables, which include centre and practice details, and number of presenting symptoms (dysuria, frequency, urgency). The patient must present with at least one of these in order to be eligible for the trial. Once all relevant data has been entered, the randomisation programme will provide the trial allocation for that participant (either Standard care or POCT arm). This information will be recorded on the baseline CRF.

There is no credible blinding mechanism that can be utilised in this trial. Immediately after receiving the allocation, the clinician will need to be aware of the result of the randomisation so as to know whether to use the point of care test or not, and the participant will be need to made aware as they may need to receive a new prescription 24 hours after initial diagnosis, once the POCT results are made available to the clinician.

#### Clinical examination

Following randomisation, participating clinicians will be asked to record details of the patient’s presenting clinical features (signs and symptoms) using a scale of 0-6 for each feature. This CRF also asks the clinician to record: the use of any diagnostic tests (i.e. urine dipstick testing), their antibiotic management for the suspected UTI (including name, dose, and duration of any medication prescribed), as well as any other medication which is either prescribed or advised. The CRF also records if any follow up appointments have been made and if the patient has taken any time off paid employment due to their symptoms.

#### POCT (flexicult) test result CRF – POCT arm participants only

For patients randomised to the POCT strategy, the result of the test should be available 24 hours after inoculation with a urine sample at the baseline consultation. The results of the test and management decisions based on results of the test will be recorded. The time of inoculation will be recorded to ensure that the test is read within 24 hours. The bacterial growth (i.e. no growth, pure growth of an organism or mixed growth, and if mixed growth then presence of predominant growth) will be recorded. The clinician will record number of colonies, the colour of colonies for bacterial identification and the antibiotic resistance of the pure or predominant. The antibiotic resistance will only be completed if the growth is ≥10^3^ cfu/mL. The clinician will then record their management decision after reading the test. This will include if they have prescribed any new antibiotic (including name, dose, and duration). The used POCT will also be photographed using standardised techniques.

### Patient follow-up

#### Symptom diary

Participants in both trial arms will be followed up on the course of their illness and their quality of life, using a two week self-completed daily symptom diary. Participants will be asked on the day that they see their GP to complete questions regarding prior UTIs and whether they had tried to use any products for their symptoms before going to see their GP. Participants will be asked to complete The Patient Enablement Instrument which rates how they are able to cope with and understand their illness [9,10]. On each of the 14 days since seeing their GP, participants will be asked to rate the presence and severity of each symptom using a scale of 0 (No problem) to 6 (Could not be worse) and will also be asked to record any antibiotic use over the 14 days. On day 14, participants are asked to complete a section of the diary which asks if they have seen any healthcare professionals regarding their UTI in the 14 days after seeing their GP.

Participants who do not return their diary will be telephoned by the trial team in order to provide a reminder and enquire whether they need any assistance in returning their diary (new postage-paid envelope for example) or given the option of giving the information required over the phone (minimum data set).

#### Resource use

Resources used in training professionals will be recorded. For POCT patients, all staff time taken to perform the test and to contact the patient to report results will be monitored. For all participants, resource use associated with UTI, including use of antibiotics, non-prescription medicines, hospitalisations, accident and emergency visits, contacts with specialist and time off work will be recorded.

#### Three-month notes review

The primary care medical records for all participants will be examined for the three months following recruitment. Data will be recorded onto paper CRFs and entered onto the online research database. Data collected will include consultations with both primary and secondary care professionals, recurrence of UTI in the three month period following recruitment and whether any antibiotics were prescribed. The results of any urine samples sent for routine laboratory culture will also be recorded.

#### Processing of samples by microbiology laboratories

Urine and stool samples obtained from all patients are analysed in the research laboratories for presence, quantity and identification of significant Gram-negative bacteria and antibiotic susceptibility profiles. All laboratories will be provided with a microbiology manual and standard operating procedures. Data will be recorded onto a standardised spreadsheet provided to each laboratory.

#### Urine processing

In all sites, urine samples from patients in both trial arms will be sent to a pre-identified research microbiology laboratory. Urines will be processed according to the POETIC microbiology manual provided and local standard operating procedures, where necessary.

Urine microscopy will be performed using standard local procedures. Culture will be performed on neat and diluted urine (10^-3^, 10^-6^) spiral-plated onto Columbia blood agar (CBA) (for total colony counts) and UTI Chromogenic agar (for species specific counts). Enumeration of bacteria will be performed 18-24 hours following incubation at 35-37°C. Bacterial counts will be summarised into purity of growth (pure, predominant, mixed 2 organisms & >2 mixed organisms).

Pure or predominant organisms from positive urines will be identified initially with chromogenic agar and confirmed using MALDI-ToF or other suitable laboratory methods.

For all UTI positive samples, organisms present at ≥10^3^ CFU/mL will be stored on cryogenic beads at -80°C. For all samples a sweep of any growth on the CBA plate will be stored on cryogenic beads at -80°C.

Susceptibility testing of positive isolate will be performed following regional research laboratory procedures, in addition, all positive isolates will also sent to the UK research laboratory for additional testing.

#### Stool processing

Stool samples will either be tested on the day of arrival at the laboratory or batched in the fridge until processed.

Dilutions of the stool sample (10^-3^ and 10^-6^) will be spiral plated onto the following antimicrobial screening plates: CBA, UTI Chromogenic agar, CRE media, ESBL media and agar supplemented with the antibiotics ciprofloxacin and gentamycin. Plates will be incubated aerobically for 18-24 hrs at 35 ± 1°C. Only the 3 most predominant species will be counted. Predominant gram negative organisms will be identified using basic microbiology or MALDI-ToF if available.

Isolates will be stored on cryogenic beads at -80°C. For all samples a sweep of any growth on the CBA plate will be stored on cryogenic beads at -80°C.

Susceptibility testing of positive isolate will be performed following regional research laboratory procedures in addition all isolates will also sent to the UK research laboratory for additional MIC testing.

### Analysis

#### Sample size calculation

A total of 614 participants (approximately 154 per network; 307 per arm) are required. We aim to improve appropriate antibiotic prescribing by at least 20% from an expected level of 55%. However, our sample size calculation is based on detecting a difference of 15 percentage points difference from control (i.e. from 55% to 70%). We need a total sample of 460 (230 for each arm) patients for final analysis (significance level α = 0.05, statistical power (1-β) = 0.90) and will recruit 614 patients to allow for a 25% loss to follow up (which requires a first urine and also follow-up via diary or telephone interview).

#### Statistical analysis

The primary outcome will be appropriate antibiotic use on day 3 (with day 1 being the day that the patient consulted with their primary care clinician). For women who are shown to have a UTI, appropriate antibiotic use on day 3 will be defined as use of an antibiotic for which the isolated pathogen has been shown to have in vitro sensitivity (based on laboratory analysis). For women who do not have a UTI, appropriate antibiotic use on day 3 will be defined as no antibiotic use on this day.

The analysis of this primary outcome will be intention-to-treat and will be undertaken utilising a multi-level logistic regression. Whilst this is an individually randomised controlled trial we will use multi-level modelling with patient as the 1^st^ level and practice as the 2^nd^ level, plus we will test for clustering at the clinician level as the outcome could be seen as a clinician behavioural measure. Country will be used as a fixed level covariate at the practice level.

Secondary outcomes will be analysed using multi-level logistic or linear regression as appropriate, except for symptoms/recovery (daily symptom score), which will be modelled over time to allow for the correlation structure between scores on different days.

This trial is not powered for any sub-group analysis, and no interim analysis is planned. Some exploratory sub-group analysis will be done by country, number of symptoms and recent antibiotic history, using the relevant variables to form an interaction term with arm in the model.

#### Economic evaluation

All resources will be valued using standard methods. Results will be reported as mean total cost (including the cost of the POCT for those in the POCT arm) per unit increase in appropriate antibiotic prescribing. Since appropriate prescribing may reduce total antibiotic dispensing and subsequent health care consulting, the intervention may be shown to dominate best practice usual care i.e. have lower cost and greater effect, in which case it will be unambiguously more cost effective. In the event of non-dominance (higher cost/greater effect) results will be reported in the form of an incremental cost effectiveness ratio (ICER) showing the extra cost per unit improvement in appropriate prescribing.

Uncertainty in any parameters will be assessed through a series of one way sensitivity analyses. Non-parametric bootstrapping using 10,000 replications will be applied to cost and effect data for both groups. A two-dimensional cost-effectiveness plane will show the uncertainty in costs and effects through a cost-effectiveness ellipse. A cost effectiveness acceptability curve will show the probability of the intervention having an incremental cost effectiveness ratio below a range of acceptability thresholds [11].

## Discussion

The POETIC trial will be the first to evaluate the diagnostic performance cost effectiveness of a POCT (Flexicult™) for the diagnosis and management of uncomplicated UTI in primary care. The appropriateness of antibiotic prescribing will be compared between patients managed with the aid of Flexicult™ POCT and patients managed according to guideline informed usual care. The Flexicult™ POCT offers the advantage of quantifying bacterial growth, organism identification and antibiotic sensitivities within 24 hours at the point of care, and may therefore be a useful adjunct to improving the antimicrobial management of UTI in primary care. Flexicult™ is already in everyday, widespread use in Danish primary care, despite it never have been subject to a clinical trial to determine its cost effectiveness. Few POCTs have been subjected to rigorous pragmatic clinical trials that evaluate not only diagnostic performance but also cost effectiveness using a range of outcomes including patient orientated measures.

As resources for health care are universally scarce, the economic evaluation will assess the cost of any benefits achieved through the use of the POCT. In addition to the direct costs associated with use of the technology this will include any savings (or additional costs) which result from more appropriate diagnosing of UTI. This will provide additional important information to health care providers and funders to aid them in making the most efficient use of their finite resources.

The analyses proposed in POETIC will generate much needed evidence that should therefore reduce equipoise about the use of this POCT in managing a common condition. If shown to be cost effective, the use of this POCT could benefit individual sufferers and provide evidence for health care authorities to develop evidence based policies to combat the spread and impact of the unprecedented rise of infections caused by multi-drug resistant-gram negative bacteria (MDR-GNB) in Europe. If shown to be not clinically and cost effective, then that too will provide evidence to change practice where it is currently used, and re-focus research efforts on developing alternative pathways for improving the management of patients presenting in primary care with symptoms attributable to UTI.

## Abbreviations

CFU/mL: Colony Forming Units per millilitre
CI: Confidence interval
CRF: Case report form
GCP: Good clinical practice
GP: General practitioner
MALDI-TOF: Matrix assisted laser desorption/ionization - time of flight mass spectrometry
MIC: Minimum inhibitory concentration
MSU: Mid-stream urine
OR: Odds ratio
PIL: Participant information leaflet
POCT: Point of care test
QoL: Quality of life
REC: Research Ethics Committee
SOP: Standard operating procedure
UKECA: United Kingdom Ethics Committee Authority
UTI: Urinary tract infection
